# Study on the influence of intelligent human–computer interaction of AI virtual anchors on consumers’ initial trust and value co-creation behavior under the technophobia

**DOI:** 10.3389/fpsyg.2025.1732258

**Published:** 2026-01-12

**Authors:** Linling Zhong, Yong Wang, Zhikun Yue, Yongzhong Yang

**Affiliations:** 1 School of Literature and Journalism, Xihua University, Chengdu, Sichuan, China; 2 School of Economics and Management, Southwest Jiaotong University, Chengdu, Sichuan, China; 3 Chengdu XYWell Technology Co., Ltd, Chengdu, Sichuan, China; 4 College of Management, Sichuan Agricultural University, Chengdu, Sichuan, China; 5 Business School, Sichuan University, Chengdu, Sichuan, China

**Keywords:** AI virtual anchors, initial trust, intelligence, technophobia, value co-creation behavior

## Abstract

**Introduction:**

In the context of the deep integration between artificial intelligence (AI) and e-commerce live streaming, virtual anchors have become a core interactive medium for both enterprises and consumers. However, when consumers first encounter e-commerce virtual anchors, the establishment of initial trust and the triggering of value co-creation behaviors are significantly influenced by psychological factors such as technophobia. Value co-creation behaviors encompass two dimensions: participation behavior and citizenship behavior. Technophobia in this context specifically manifests as concerns about the unknown risks of AI technology, operational complexity, and resistance to non-humanized interactions.

**Methods:**

Based on the Technology Acceptance Model (TAM), this study develops a mechanism to examine how the intelligence of human-machine interaction in e-commerce virtual anchors influences consumers’ initial trust and value co-creation behaviors, from the perspective of technophobia. To validate this model and related hypotheses, we conducted a specialized survey targeting consumers with initial exposure to e-commerce virtual anchors, collected 337 valid responses, and used Structural Equation Modeling (SEM) to test 14 research hypotheses.

**Results:**

The results showed that 12 hypotheses were confirmed. Specifically, the four dimensions of intelligence possessed by e-commerce virtual anchors—guidance, recognition, analysis, and feedback—all positively influence perceived usefulness and perceived ease of use. These two perceived variables further positively promote the formation of consumers’ initial trust. Regarding the impact of initial trust on value co-creation behaviors, it significantly drives consumer participation behaviors (e.g., asking questions, posting comments, following livestreaming room rules) but shows no significant effect on consumers’ citizenship behaviors (e.g., actively promoting livestreaming rooms, reporting service defects, assisting other consumers). Meanwhile, technophobia plays a significant negative moderating role in the relationship between perceived ease of use and initial trust. Two hypotheses were not supported: first, initial trust does not significantly drive citizenship behaviors, potentially due to negative cognitive legacy from early low-intelligence virtual anchors; second, technophobia does not significantly moderate the relationship between perceived usefulness and initial trust, as it primarily stems from concerns about operational complexity rather than doubts about utility.

**Discussion:**

The findings clarify the boundary role of technophobia, particularly its key disruptive effect in the “perceived ease of use → initial trust” transformation process, thereby enriching the application dimensions of TAM in human-computer interaction scenarios. The core value of this study lies in providing empirical evidence for e-commerce enterprises to effectively build consumers’ initial trust and promote differentiated value co-creation through measures such as alleviating technophobia and optimizing virtual anchor interaction design.

## 1 Introduction

In recent years, AI technology has driven significant transformations in the live streaming e-commerce industry (Liu and Chen, 2025). AI virtual anchors have gradually become an important force replacing human anchors due to their advantages, such as low investment costs, high stability, and freedom from restrictions on live streaming duration and location (Santhoshkumar et al., 2023). AI virtual anchors are widely used in product introductions, service provision, and shopping assistance, serving as a new interactive medium connecting enterprises and consumers (Zhang et al., 2025). iResearch Consulting Group (2023) points out that AI virtual anchors will enter a stage of rapid, refined development in the future. However, the virtual nature of e-commerce transactions, combined with unfamiliarity with human–computer interaction, makes “initial trust” a core barrier to consumers establishing connections with virtual anchors. Particularly during consumers’ first interactions with AI virtual anchors, they lack awareness of the intelligent responsiveness and service reliability of these systems. It may lead to negative perceptions due to perceived lack of intelligence in virtual anchors or to a refusal to build trust out of fear of AI technologies (Ilona et al., 2018), ultimately making it difficult to trigger value co-creation behaviors.

From a theoretical perspective, the TAM has been validated as a practical framework for explaining consumer adoption mechanisms of new technologies. Its core variables (perceived usefulness and perceived ease of use) serve as key determinants in trust formation (Davis, 1989; Hampton-Sosa and Koufaris, 2005). However, existing research predominantly focuses on trust mechanisms in e-commerce platforms or human live streamers. For AI virtual anchors as “dynamic AI interaction entities” in e-commerce, there remains a lack of clarity regarding how their intelligence influences initial trust through perceptual variables, particularly in systematically investigating the transmission path of “intelligence, perception, initial trust” during first-contact scenarios.

Moreover, technophobia plays a critical role in the formation of initial trust during first-contact interactions. Technophobia refers to negative psychological responses (e.g., anxiety, rejection) arising from unknown risks, concerns about operational complexity, and resistance to non-human interactions when encountering emerging technologies such as AI (Bin and Yan, 2020). Consumers unfamiliar with the logic of virtual anchor interaction may experience behavioral hesitation due to concerns about “operational complexity concerns.” Whether such hesitation directly hinders the establishment of initial trust and consequently affects value-creation behavior remains to be empirically validated. Notably, value co-creation behavior encompasses two dimensions: participation behavior (e.g., low-threshold interactions like liking, asking questions, and following livestream rules) and citizenship behavior (e.g., active promotion, reporting service defects, and assisting other consumers). The driving effects of initial trust on these two dimensions may differ, necessitating theoretical research to clarify their underlying mechanisms.

Practical applications reveal that some e-commerce companies overlook the importance of building initial trust during consumers’ first interactions with AI virtual anchors. Although their virtual anchors exhibit basic intelligent capabilities, these systems are frequently discontinued because users struggle to recognize their ease of use and practical value quickly. Additionally, businesses fail to address consumer technophobia by implementing effective strategies to alleviate resistance toward virtual technologies, ultimately preventing the transition from passive viewing to active participation in co-creation (Jia, 2019). Therefore, clarifying the mechanisms of initial trust formation and the moderating role of technophobia during consumers’ first encounter with AI virtual anchors has become a critical issue for addressing industry pain points and promoting the healthy development of the virtual anchor ecosystem.

## 2 Literature review

### 2.1 Technology acceptance model

The model suggests that when users encounter new technologies, they assess both perceived usefulness and perceived ease of use to determine whether to adopt them early (Davis, 1989). These two psychological factors are key determinants of user acceptance. Perceived ease of use refers to the degree to which an individual believes that using a particular technology would be free of effort. In contrast, perceived usefulness indicates the extent to which users believe the technology can enhance their job performance (Yoon and Lee, 2021).

Scholars have extended the application of the TAM in e-commerce by integrating the unique characteristics of online shopping environments with the concept of consumer trust. Empirical studies have validated several models that combine e-commerce trust with technology acceptance frameworks. For instance, Gefen and Straub (2003) proposed a model that integrates the TAM with theories of trust formation in e-commerce. He argued that external factors in e-commerce influence perceived usefulness and perceived ease of use, which in turn affect consumer trust. This trust subsequently shapes consumers’ attitudes and behaviors toward online shopping.

### 2.2 AI virtual anchors

Artificial intelligence virtual anchors are digital avatars created through AI technology that integrate voice systems and motion recognition to replace human anchors (Shi and Yue, 2023). Advanced AI capabilities and extensive speech databases enable these virtual anchors to perform anchor duties effectively (Kaplan and Haenlein, 2019), thereby driving significant industry transformation. In live streaming, they are gradually replacing human anchors (Santhoshkumar et al., 2023). AI virtual anchors offer lower investment costs and greater stability than human anchors, and are not constrained by broadcast duration or location (Niu et al., 2023). From a service perspective, Dong et al. (2023) notes that virtual anchors possess appeal and novelty, naturally generating traffic through topic engagement, thereby boosting livestream performance. From an industry development perspective, AI virtual anchors can address the “anchor dilemma” faced by human live streamers in e-commerce. On the one hand, human anchors may face reputational damage; on the other hand, the uneven quality of human anchors makes it difficult for e-commerce companies to recruit suitable candidates. It highlights a key direction for the future development of e-commerce live streaming (Zhong et al., 2024).

### 2.3 Intelligence

Intelligence is the fundamental attribute of virtual humans, endowed by AI technology (Slota et al., 2023). Virtual humans perceive their internal and external environments through intelligent technologies and execute actions with inherent intelligence (Orozco et al., 2011). Their intelligent behaviors reflect psychological states and simulate emotional characteristics such as mood (Vinayagamoorthy et al., 2006), enabling them to interact with their surroundings while possessing feedback-generating capabilities that influence them (Herrero and Antonio, 2005). As virtual human intelligence continues to evolve, new economic production models are emerging. As representatives of AI, virtual humans have become labor subjects participating in the division of labor, enhancing productivity and presenting new opportunities for comprehensive human-society development (Su and Guo, 2019). To better serve users, developers integrate more human-like intelligent behaviors into virtual human programs through algorithms (Li, 2003), thereby improving their intelligence for seamless interaction in application scenarios (Pan et al., 2007). Zhong et al. (2025) conducted grounded research revealing that the intelligence of e-commerce virtual anchors comprises four dimensions: guidance intelligence, recognition intelligence, analysis intelligence, and feedback intelligence. The study developed corresponding measurement scales and validated their scientific validity through empirical research.

### 2.4 Initial trust

Trust forms the foundation of exchange behaviors (Granovetter, 1973). In e-commerce contexts, trust refers to consumers’ expectations regarding the performance perceptions of products and services (Le, 2015). Gu (2022) posits that consumer trust constitutes the core competitive advantage in livestreaming commerce, where trust in anchors and products significantly influences purchasing decisions (Wang, 2023). With AI technology increasingly integrated into various interactive scenarios, scholars have expanded trust research to the human-machine relationship level, exploring the logic behind users’ trust in AI. Reyes et al. (2025) found through game experiments that visualizing the uncertainty of AI predictions can effectively enhance user trust. Zarifis and Cheng (2024), focusing on financial consulting scenarios, proposed that trust-building in generative AI requires adaptation to problem types—AI’s human-like interaction features can strengthen trust when addressing ambiguous financial questions.

Initial trust—the foundational form of trust—shapes future relationships between communicators (McKnight et al., 2002). This initial assessment occurs during anchors’ first contact with audiences and is based on an evaluation of their comprehensive capabilities (Shi and Li, 2021). As the first step in trust-building, it establishes an enduring foundation for sustained relationships (Audun et al., 2006). In e-commerce, establishing initial trust is critical to consumer decisions and platform sustainability. The virtual nature of transactions introduces heightened uncertainty and credit risks, making initial trust acquisition particularly vital for digital commerce (Salo and Karjaluoto, 2007). Research on initial trust in AI interaction scenarios further reveals unique formation mechanisms. Yanzeng et al. (2025) found that bubble coloring designs in chatbots enhance information processing fluency, strengthening users’ initial trust in AI and, consequently, increasing their willingness to disclose themselves. Dao et al. (2025), using Vietnamese Gen Z consumers as the sample, found that in medical AI contexts, the “traditional self” in individual self-concept negatively affects initial trust, whereas the “modern self” positively influences it. That initial trust directly promotes willingness to adopt AI.

### 2.5 Value co-creation behavior

In management studies, the “value co-creation” approach manifests through consumer experiences, where businesses create value by providing exceptional consumer interactions (Prahalad and Ramaswamy, 2004). Throughout this process, consumers act as value creators while enterprises serve as co-creators through service engagement (Grönroos, 2008). Yi and Gong (2013) categorized consumer value co-creation into two dimensions: participation behavior and citizenship behavior. Participation behavior includes information seeking (collecting external information to meet needs), information sharing (discussing known information with others), compliance behavior (adhering to rules and instructions), and interpersonal interaction (engaging with others). Citizenship behavior encompasses feedback (providing suggestions for long-term development), advocacy (promoting products/services), altruism (helping peers), and tolerance (accepting others’ mistakes).

### 2.6 Technophobia

In psychology, “fear” is considered one of the most primal human emotions, arising from our self-protective mechanisms in response to unfamiliar environments or potential threats (Wang and Kong, 2023). Technophobia analyzes the thoughts or behaviors of users who develop negative emotions, such as anxiety and fear, towards computers and thus destroy or resist them (Bin and Yan, 2020). As research progressed, scholars broadened the construct from computer-specific anxiety to encompass apprehension toward emerging technologies in general (Aksoy et al., 2020).

Technophobia towards AI is relatively common among people. In an online survey conducted by the British Science Association, 60% of respondents believed AI would reduce employment opportunities within a decade, and 36% viewed AI development as a threat to humanity’s long-term survival (Oh et al., 2017). Scholarly research on various AI systems identifies two primary sources of technophobia: First, the perceived greater-than-anticipated job displacement—where AI’s superior productivity might replace significant numbers of workers (Mills, 1997)—has fueled fears of being replaced by AI (Xu and Song, 2022). Second, uncertainties surrounding continuous technological advances, particularly the fear that AI could attain autonomous consciousness and pose existential risks to humanity (Han and Zhao, 2020).

## 3 Research hypothesis and research model

The TAM is widely regarded as an effective tool for assessing audience adoption of emerging technologies (Chen et al., 2020). Accordingly, this study extends TAM by treating AI virtual anchor intelligence as an external variable and examining how it affects consumers’ value co-creation behaviors through the mediating mechanism of initial trust. Based on the intelligent performance of virtual anchors in human-computer interactions during service delivery, Zhong et al. (2024) categorized virtual anchor intelligence into four dimensions: guidance intelligence, recognition intelligence, analysis intelligence, and feedback intelligence. This study explores the impacts of these four dimensions on perceived usefulness and perceived ease of use. Previous studies have found that perceived usefulness and perceived ease of use further influence initial trust (Hampton-Sosa and Koufaris, 2005), which ultimately shapes consumers’ value co-creation behaviors.

Sang (1999) proposed that trust transmission must account for human subjectivity. The trust transmission theory emphasizes that the sender’s subjective perception serves as a crucial boundary condition, shaping the effectiveness of trust transfer. When encountering new technologies, consumers may develop subjective fears, leading to psychological resistance or rejection of these innovations—a phenomenon referred to as technophobia. Similarly, during their first interaction with AI virtual anchors, consumers might experience anxiety toward virtual human technology, resulting in subconscious resistance to these digital avatars.

### 3.1 The rationale and validity of separate testing for the four dimensions of e-commerce virtual anchors’ intelligence

This study conducts independent testing of the four sub-dimensions of e-commerce virtual anchors’ intelligence (guidance intelligence, recognition intelligence, analysis intelligence, and feedback intelligence), based on the following core rationale: First, the sub-dimension classification is grounded in robust empirical evidence and literature support. Zhong et al. (2025) employed grounded research methods to deeply immerse in real-world human-computer interaction scenarios of e-commerce virtual anchors, systematically refining and validating these four core sub-dimensions. It confirms that the classification accurately aligns with consumers ‘perception of virtual anchors’ intelligence performance, representing empirical induction from practical scenarios rather than subjective categorization. It provides a fundamental theoretical basis for testing the impact of each dimension separately.

Secondly, the functional orientations and operational pathways of each sub-dimension exhibit fundamental differences. Guidance intelligence emphasizes proactive service adaptation; recognition intelligence focuses on precise demand capture; analysis intelligence prioritizes problem-solving efficiency; while feedback intelligence concentrates on interactive response quality. Merging these dimensions into a higher-order factor would only validate the influence of “overall intelligence,” failing to reveal the differentiated impacts of the various intelligence dimensions on perceptual variables and initial trust, and thereby losing the ability to identify core influence pathways accurately.

Finally, the independent testing aligns with the study’s practical value orientation. One of the core objectives of this research is to provide actionable optimization strategies for relevant enterprises. By separately evaluating the effectiveness of each sub-dimension, it directly clarifies the optimization priorities for businesses. This precise guidance value is unattainable with high-order factor models, which can only demonstrate “overall effectiveness of intelligence” but fail to address the core question: “which types of intelligent capabilities enterprises should prioritize for optimization.”

### 3.2 Mediating role of initial trust

This study establishes the core mediating role of “initial trust” between perceived usefulness and perceived ease of use and value co-creation behavior. This framework serves as the key logical pivot in the model, as demonstrated below: From the perspective of theoretical transmission logic, the TAM fundamentally operates through “perceived variables → attitude/behavior” transformation (Davis, 1989). In human-computer interaction contexts, trust serves as the essential bridge connecting cognitive evaluation to behavioral decision-making (McKnight et al., 2002). While perceived usefulness reflects consumers ‘assessment of e-commerce virtual anchors’ “practical value” and perceived ease of use evaluates “operational convenience,” these evaluations do not directly trigger value co-creation. Consumers must first establish initial trust in the anchors’ reliability and service capabilities through these perceptions, which then eliminates interaction concerns and facilitates the transition from “cognitive recognition” to “participation behavior.” Therefore, the core transmission path in this study is: E-commerce virtual anchor intelligence → Perceived usefulness/Perceived ease of use → Initial trust → Value co-creation behavior. Given the unique nature of the research scenario, in which e-commerce virtual anchors function as “dynamic AI interaction agents” lacking offline physical support, the mediating role of initial trust is particularly critical. In first-time contact scenarios, consumers lack understanding of virtual anchors’ service quality and response reliability. While perceived usefulness and perceived ease of use can lower the “technical acceptance threshold,” initial trust further reduces “uncertainty in virtual transactions,” motivating consumers to engage in participation behaviors such as liking, asking questions, and even proactively promoting or reporting flaws. Existing research confirms that in AI-driven human-computer interactions, trust serves as the core mediator transforming perceived value into behavioral intention (Kaplan and Haenlein, 2019). Without this intermediary, the explanatory power of perceived variables on co-creation behaviors would significantly weaken. From a model rigor perspective, this study clarifies the mediating role of initial trust, avoiding the logical leap that “perceived usefulness/perceived ease of use directly impacts co-creation.” In prior studies on e-commerce trust, Gefen and Straub (2003) established that perceived variables influence consumer behavior through trust, while Hampton-Sosa and Koufaris (2005) further highlighted the pivotal mediating role of initial trust in first-contact scenarios. This research builds upon this theoretical framework to align variable relationships with the practical logic of consumer decision-making.

### 3.3 Specific research hypotheses

#### 3.3.1 The influence of AI virtual anchors’ intelligence on perceived ease of use and perceived usefulness

As AI-powered entities, AI virtual anchors can perceive and utilize surrounding information while identifying consumers’ genuine needs (Szewcyzk et al., 2009). These digital assistants effectively channel consumer demands and resolve issues with higher precision. The higher the intelligence level of AI virtual anchors, the more efficiently they can provide effective services—consumers do not need to perform additional operations, as simply stating their requirements allows these intelligent systems to interpret basic requests accurately. AI virtual anchors gather consumer insights through human-computer interactions and analyze data to provide customized solutions (Yang and Wang, 2021), thereby enhancing consumer service quality (Abraham et al., 2018). Fortunati and Edwards (2020) proposed that with the advancement of AI technology, audiences can communicate with AI entities as easily as they do with friends; the improved intelligence of AI virtual anchors enables consumers to interact with them more effortlessly.

In conclusion, this study proposes the following hypotheses:

*H1*: Guidance intelligence positively affects perceived ease of use.

*H2*: Recognition intelligence positively affects perceived ease of use.

*H3*: Analysis intelligence positively affects perceived ease of use.

*H4*: Feedback intelligence positively affects perceived ease of use.

*H5*: Guidance intelligence positively affects perceived usefulness.

*H6*: Recognition intelligence positively affects perceived usefulness.

*H7*: Analysis intelligence positively affects perceived usefulness.

*H8*: Feedback intelligence positively affects perceived usefulness.

#### 3.3.2 The influence of perceived ease of use and perceived usefulness on consumers’ initial trust

Gefen and Straub (2003) posited that in e-commerce contexts, perceived ease of use and perceived usefulness are critical determinants of consumer acceptance, positively influencing initial trust. Lu and Zhou (2007) further demonstrated that these two dimensions reflect the positive shopping experience provided by e-commerce platforms, which helps establish consumer trust. Kucukusta et al. (2015) identified their significant positive impact on the formation of initial trust. Empirical research by Lv and Cao (2021) revealed that in social commerce environments, both perceived usefulness and perceived ease of use positively influence the development of initial trust.

In conclusion, this study proposes the following hypotheses:

*H9*: Perceived ease of use positively affects consumers’ initial trust.

*H10*: Perceived usefulness positively affects consumers’ initial trust.

#### 3.3.3 The influence of consumers’ initial trust on consumers’ value co-creation behaviors

When consumers first encounter AI virtual anchors, they evaluate the anchors’ overall performance to determine whether to grant initial trust. Initial trust represents the foundational state of trust (McKnight et al., 2002). The establishment of initial trust between trust givers and trust takers facilitates the formation of collaborative relationships (Shi and Li, 2021). In online shopping scenarios, trust serves as a crucial bridge between consumers and e-commerce platforms, and initial trust significantly influences consumer behavior (Zhang and Yuan, 2020). Yi and Gong (2013) posits that consumers’ value co-creation behaviors can be categorized into participation behaviors and citizenship behaviors. When consumers initially trust AI virtual anchors, they are more likely to engage in interactive activities (e.g., asking questions) and to provide mutual assistance when other consumers request help.

In conclusion, this study proposes the following hypotheses:

*H11*: Consumers’ initial trust positively affects consumers’ participation behaviors.

*H12*: Consumers’ initial trust positively affects consumers’ citizenship behaviors.

#### 3.3.4 The moderating effect of technophobia on the relationships between perceived ease of use, perceived usefulness, and consumers’ initial trust

Technophobia refers to negative psychological states (e.g., anxiety, resistance) triggered by uncertainty, concerns, and stress when humans encounter emerging technologies (Bin and Yan, 2020). McKnight (2004) found that individuals’ perceptions of personal computers significantly influence their overall trust in e-commerce. In practice, many audiences also worry about and fear the impact of virtual humans on real life, leading them to take steps to resist their application (Jia, 2019). When consumers first encounter AI virtual anchors, they are not yet familiar with the application of virtual human technology in e-commerce live streaming. Moreover, factors such as the uncertainty of emerging technologies, feelings of anxiety, and stress may trigger technophobia. Technophobia can make it difficult for consumers to trust AI virtual anchors, leading to negative experiences and thereby affecting the process from consumers’ acceptance of new technologies to the formation of their initial trust. Therefore, this study treats technophobia as a boundary condition that moderates the relationships among perceived usefulness, perceived ease of use, and initial trust.

In conclusion, this study proposes the following hypotheses:

*H13*: Technophobia negatively moderates the relationship between perceived ease of use and consumers’ initial trust.

*H14*: Technophobia negatively moderates the relationship between perceived usefulness and consumers’ initial trust.

**Figure 1:**
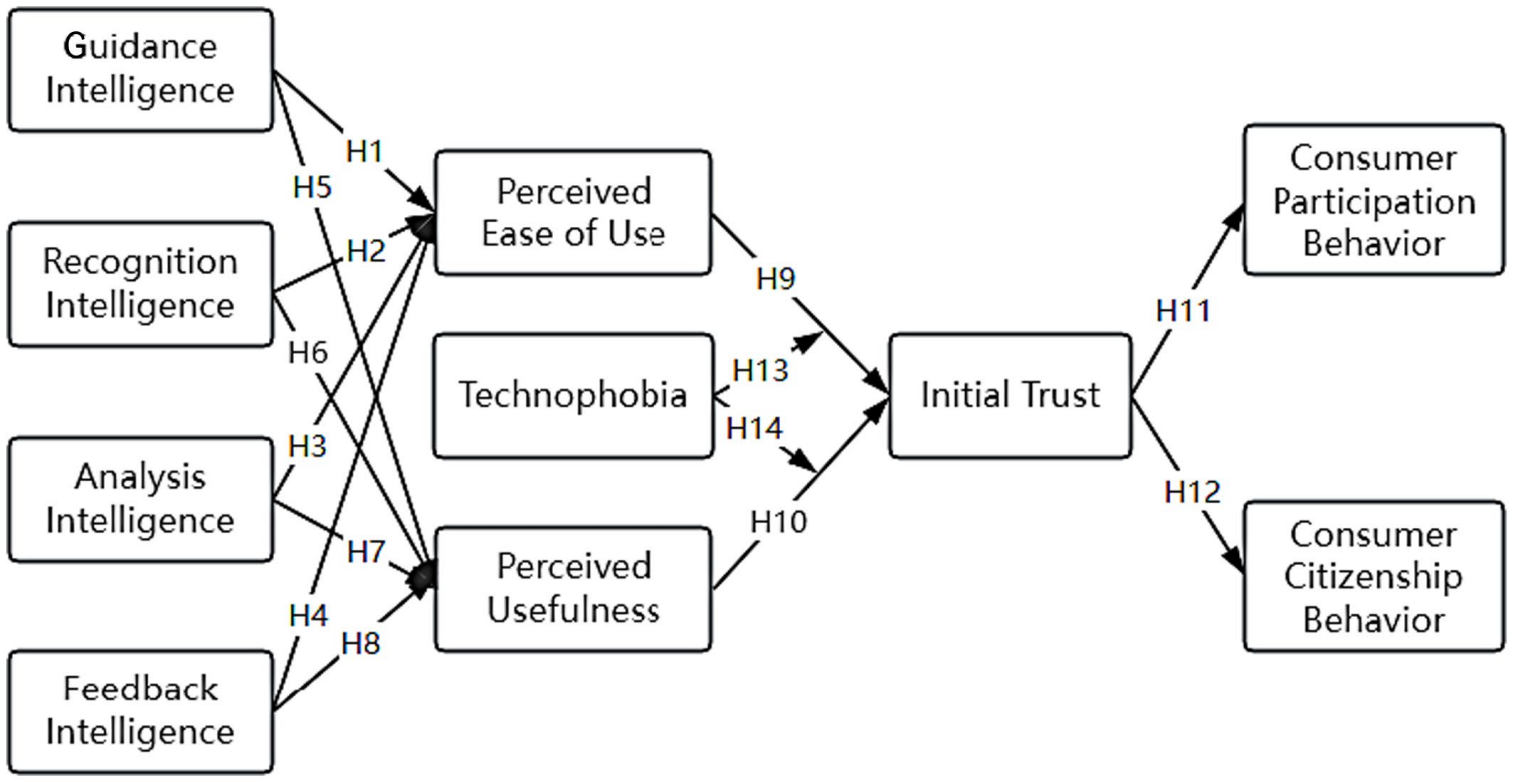
The research model.

Based on the above hypotheses, Figure 1 shows the research model constructed in this study.

## 4 Research method

### 4.1 Scale design

#### 4.1.1 Questionnaire content

The questionnaire in this study comprises three main sections:

1. Introduction and informed consent form. We begins by providing a self-introduction and explaining the research purpose and the intended use of the questionnaire. Participants are clearly informed that the collected data will be used exclusively for academic research, with strict confidentiality of personal information to ensure their voluntary participation and genuine intent. The section concludes with sincere gratitude to all participants.
2. Collection and screening of participants’ basic information. The questionnaire included five demographic items: gender, age, education level, and occupation to collect participants’ basic information. Additionally, since initial consumer trust correlates with the frequency of exposure to e-commerce virtual anchors and develops during the first encounter with such anchors, eligible participants were required to have watched their livestreaming sales for the first time. To ensure clear impressions of this initial experience, the questionnaire added a screening item: “Have you recently watched e-commerce virtual anchors’ livestreaming sales for the first time?” Participants who answered “No” would be directed to “End Questionnaire,” while those who answered “Yes” could proceed. Simultaneously, we included a verification item: “This question checks your attentiveness. Please select 2 [single choice]” to confirm active participation.
3. Measurement scale for the variable. Before presenting the scale items, clearly explain the core variable to participants: “In this questionnaire, ‘E-commerce Virtual anchor Intelligence’ refers to the interactive capabilities demonstrated by virtual anchors you engage with, including guidance services (e.g., proactively recommending suitable products), demand recognition (e.g., understanding your inquiry intent), decision-making analysis (e.g., providing suggestions based on your needs), and responsive feedback (e.g., timely and relevant replies).” It helps participants accurately understand the measurement dimensions. The questionnaire in this study included demographic information items to collect participants’ basic information. The scale for the variable “intelligence of AI virtual anchors” was derived from Zhong et al. (2025); the scales for “perceived ease of use” and “perceived usefulness” were adapted from Davis (1989); the scale for “initial trust” came from McKnight et al. (2002); and the scale for “technophobia” was adopted from Khasawneh (2018). All questionnaires used a 7-point Likert scale, where scores 1–7 represent participants’ degree of agreement—for example, “1 represents strongly disagree” and “7 represents strongly agree”.

#### 4.1.2 Preliminary research

To ensure the scientific validity and applicability of the variable measurement scales, this study first established a pre-survey team comprising 30 professors and master’s/doctoral students in the management field, all of whom were invited to participate. All team members had experience of watching AI virtual anchors live streams and shopping in e-commerce live stream rooms, which ensured both professional expertise and scenario adaptability.

We conducted the pre-survey in three stages. First, we refined the wording of the measurement scale by discussing and revising vague or ambiguous items. Second, we collected data by asking team members to complete the scale, which produced 30 valid responses. Third, we assessed the scale’s quality and confirmed that its reliability and validity met the required standards—Cronbach’s *α* coefficients exceeded 0.7, KMO values exceeded 0.6, and factor loadings exceeded 0.5. Based on these results, we finalized the scale for use in the formal survey.

### 4.2 Data collection

A total of 400 formal questionnaires were distributed in this study: 200 through online channels and 200 through offline channels. Researchers excluded questionnaires with incorrect responses to screening items and those suspected of being completed carelessly (e.g., selecting the same option for six consecutive questions or containing blank items). Ultimately, 337 valid questionnaires were retained, with an effective response rate of 84.25%.

Table 1 presents the demographic characteristics of the participants. The sampling conformed to the audience characteristics of AI virtual anchors’ live streaming rooms.

**Table 1:** Demographic data analysis results (*N* = 337).

Essential information	Classify	Number	Proportion
Sex	Male	149	44.21%
	Female	188	55.79%
Age	18–30 years	121	35.91%
	31–40 years	153	45.40%
	41–50 years	44	13.06%
	51–60 years	17	5.04%
	Over 60	2	0.59%
Record of formal schooling	Junior high school and below	5	1.48%
	High school/vocational school/technical school	31	9.20%
	Junior college	93	27.60%
	Undergraduate course	111	32.94%
	Master’s degree or above	97	28.78%
Occupation	State-owned enterprises	82	24.33%
	Government/Institutional units	75	22.26%
	Private enterprise	72	21.36%
	Foreign-funded enterprises	53	15.73%
	Student	21	6.23%
	Liberal professions	34	10.09%
Monthly income	Under 3,000 yuan	51	15.13%
	3,000 yuan to 6,500 yuan	91	27.00%
	6,500 yuan-10,000 yuan	117	34.72%
	10,000 yuan plus	78	23.15%

This study conducted descriptive statistics on 337 valid samples using SPSS 20.0, with the results shown in Table 2. It indicates that all item standard deviations were less than 2, and the absolute values of skewness and kurtosis were all below 2—both meet the criteria for normal distribution. This result demonstrates that the sample data follow a normal distribution and are suitable for subsequent statistical tests.

**Table 2:** Descriptive statistical analysis results.

Class	Heading	Mean	Standard deviation	Variance	Skewness	Kurtosis
Sex	Q1	1.56	0.497	0.247	−0.234	−1.957
Age	Q2	1.89	0.857	0.735	0.926	0.712
Record of formal schooling	Q3	3.78	1.011	1.021	−0.442	−0.525
Occupation	Q4	2.88	1.582	2.502	0.547	−0.699
Monthly income	Q5	2.66	0.997	0.993	−0.199	−1.009
Guidance intelligence	GIN1	4.36	1.56	2.435	−0.168	−0.74
	GIN2	4.34	1.46	2.13	−0.111	−0.768
	GIN3	4.45	1.533	2.35	−0.115	−1.065
	GIN4	4.42	1.498	2.245	−0.088	−0.841
Recognition intelligence	RIN1	4.31	1.526	2.329	−0.137	−0.869
	RIN2	4.3	1.505	2.265	−0.141	−0.915
	RIN3	4.57	1.501	2.252	−0.106	−0.811
	RIN4	4.5	1.365	1.864	−0.241	−0.742
Analysis intelligence	AIN1	4.11	1.811	3.279	−0.021	−1.182
	AIN2	4.06	1.735	3.011	0.045	−0.982
	AIN3	4.14	1.76	3.099	0.038	−1.068
	AIN4	4.15	1.719	2.954	−0.002	−0.962
	AIN5	4.27	1.795	3.222	−0.174	−1.076
Feedback intelligence	FIN1	4.69	1.729	2.988	−0.444	−0.867
	FIN2	4.52	1.628	2.649	−0.09	−1.088
	FIN3	4.56	1.61	2.593	−0.131	−1.145
	FIN4	4.68	1.621	2.628	−0.155	−1.21
	FIN5	4.73	1.641	2.693	−0.336	−0.999
Perceived ease of use	PEU1	4.16	1.833	3.361	−0.127	−1.089
	PEU2	4.22	1.739	3.025	−0.083	−1.053
	PEU3	4.23	1.692	2.863	−0.163	−0.981
	PEU4	4.3	1.746	3.049	−0.151	−1.012
Perceived usefulness	PUS1	3.96	1.893	3.585	0.003	−1.189
	PUS2	4.07	1.88	3.533	−0.062	−1.209
	PUS3	4.09	1.911	3.653	−0.033	−1.237
	PUS4	4	1.908	3.64	0.025	−1.238
	PUS5	3.94	1.959	3.836	0.017	−1.267
Initial trust	INT1	4.3	1.508	2.275	−0.237	−0.609
	INT2	4.35	1.542	2.377	−0.174	−0.784
	INT3	4.33	1.454	2.114	−0.085	−0.563
	INT4	4.39	1.437	2.066	−0.341	−0.498
Consumer participation behavior	PAR1	4.14	1.697	2.878	−0.077	−0.88
	PAR2	4.13	1.683	2.832	0.028	−0.855
	PAR3	4.15	1.764	3.113	−0.2	−0.958
	PAR4	4.22	1.727	2.981	0.017	−1.066
Consumer citizenship behavior	CIT1	4.49	1.687	2.846	−0.230	−1.104
	CIT2	4.57	1.623	2.633	−0.169	−1.012
	CIT3	4.68	1.636	2.676	−0.215	−1.026
	CIT4	4.60	1.675	2.807	−0.316	−1.029

As shown in Table 3, the correlation coefficients among latent variables all remained within the standard threshold of 0.7, indicating relatively strong overall correlations. While these results provide preliminary predictive support for subsequent hypothesis testing, correlation analysis alone cannot conclusively determine quantitative dependencies between variables. Therefore, structural equation modeling should be employed in this study to validate the specific relationships among these variables further.

**Table 3:** Correlation test results between variables.

Variable	Mean	Standard deviation	GIN	RIN	AIN	FIN	PEU	PUS	INT	PAR	CIT
GIN	4.3954	1.37432	1								
RIN	4.1472	1.44879	0.252**	1							
AIN	4.4206	1.31052	0.645**	0.286**	1						
FIN	4.635	1.46558	0.603**	0.328**	0.620**	1					
PEU	4.2277	1.47027	0.508**	0.333**	0.517**	0.510**	1				
PUS	4.0125	1.60675	0.520**	0.337**	0.565**	0.533**	0.309**	1			
INT	4.3412	1.32232	0.239**	0.177**	0.371**	0.231**	0.252**	0.262**	1		
PAR	4.1588	1.43174	0.214**	0.211**	0.217**	0.231**	0.222**	0.124*	0.221**	1	
CIT	4.5846	1.52493	0.208**	0.135**	0.287*	0.302**	0.188**	0.207**	0.035	0.193**	1

**Correlation is significant at the 0.01 level, *Correlation is significant at the 0.05 level.

## 5 Results of empirical evidence

To ensure the reliability of the empirical analysis results, this study evaluates data quality across three dimensions: common-method bias tests, reliability tests, and validity tests, ensuring that the measurement tools and data samples meet academic research standards.

### 5.1 Common method bias testing

As the study’s data originate from self-reported questionnaires, it may be susceptible to standard-method bias due to a single data source. To mitigate this, we employed Harman’s widely recognized single-factor test for validation. The test’s core logic is that if significant standard method bias exists, the variance across all measurement items would be highly concentrated in a single common factor. Typically, a variance explanation ratio of less than 40% for the first common factor is considered a non-biased result.

The specific test operation is as follows: all the measurement items in the questionnaire are carried out for non-rotated exploratory factor analysis, the common factor is extracted by the principal component analysis (the extraction standard is eigenvalue>1), the orthogonal rotation is carried out by the maximum variation method, the test results are based on the total variation analysis, see Table 4.

**Table 4:** Results of total variation.

Ingredient	Initial eigenvalues			Extract square and load			Rotation square and load		
	Amount to	Percentage of variance	Accumulate %	Amount to	Percentage of variance	Accumulate %	Amount to	Percentage of variance	Accumulate %
1	12.521	32.106	32.106	12.521	32.106	32.106	3.939	10.099	10.099
2	3.278	8.406	40.511	3.278	8.406	40.511	3.857	9.89	19.99
3	3.064	7.857	48.369	3.064	7.857	48.369	3.519	9.022	29.012
4	2.78	7.127	55.496	2.78	7.127	55.496	3.498	8.969	37.981
5	2.348	6.022	61.517	2.348	6.022	61.517	3.286	8.425	46.406
6	1.977	5.068	66.586	1.977	5.068	66.586	3.258	8.353	54.759
7	1.495	3.834	70.42	1.495	3.834	70.42	3	7.692	62.451
8	1.311	3.361	73.781	1.311	3.361	73.781	2.855	7.32	69.771
9	1.064	2.727	76.508	1.064	2.727	76.508	2.627	6.737	76.508
10	0.555	1.424	77.932						
11	0.526	1.35	79.281						
12	0.492	1.262	80.544						
13	0.461	1.183	81.726						
14	0.453	1.162	82.889						
15	0.445	1.141	84.03						
16	0.418	1.072	85.102						
17	0.404	1.036	86.137						
18	0.379	0.973	87.11						
19	0.371	0.952	88.062						
20	0.359	0.92	88.982						
21	0.337	0.864	89.846						
22	0.32	0.821	90.667						
23	0.308	0.79	91.457						
24	0.303	0.777	92.235						
25	0.288	0.738	92.973						
26	0.27	0.692	93.665						
27	0.263	0.675	94.341						
28	0.26	0.667	95.008						
29	0.242	0.62	95.628						
30	0.219	0.562	96.19						
31	0.21	0.538	96.728						
32	0.202	0.517	97.246						
33	0.188	0.483	97.729						
34	0.174	0.446	98.174						
35	0.167	0.429	98.604						
36	0.159	0.408	99.011						
37	0.15	0.384	99.395						
38	0.138	0.353	99.748						
39	0.098	0.252	100						

Based on the total variance results in Table 4, the single-factor analysis by Harman yields the following conclusions: First, nine common factors with eigenvalues greater than one were identified, indicating that no single factor dominates all item variance and preliminarily excluding the possibility of severe standard-method bias. Second, the initial variance explained by the first common factor (32.106%) is significantly below the 40% threshold, suggesting that item variance is not concentrated in a single factor and standard method bias has minimal interference. Third, the cumulative variance explained by the nine common factors reaches 76.508%, demonstrating complete factor extraction and statistically valid results. In summary, the data in this study show no significant standard-method bias, providing a solid foundation for subsequent empirical analysis.

### 5.2 Reliability and validity tests

The KMO value for this study is 0.924, which exceeds the conventional minimum threshold of 0.7. Meanwhile, the approximate chi-square value of Bartlett’s sphericity test reaches 9706.383, with corresponding degrees of freedom at 741, and the significance level (sig) is 0.000, lower than the critical test value of 0.05. This study conducted commonality tests on the sample data, with all item commonalities exceeding 0.6. The above test results indicate that the questionnaire data collected in this study exhibit good construct validity of *Broussonetia papyrifera* and are suitable for subsequent factor analysis.

The CITC and reliability analysis results of this study are shown in Table 5. The Cronbach’s *α* values for all scales are above 0.7, while the item-specific CITC values remain below the 0.35 threshold. Notably, removing any single item would lower Cronbach’s α below the original scale’s level, indicating good reliability and high internal consistency.

**Table 5:** Reliability analysis results.

Variable	Heading	CITC	Cronbach’s α after deletion of items	Cronbach’s α
Guidance intelligence	GIN1	0.826	0.911	0.929
	GIN2	0.85	0.903	
	GIN3	0.824	0.911	
	GIN4	0.838	0.906	
Recognition intelligence	RIN1	0.742	0.904	0.911
	RIN2	0.783	0.89	
	RIN3	0.868	0.858	
	RIN4	0.803	0.884	
Analysis intelligence	AIN1	0.709	0.854	0.879
	AIN2	0.694	0.857	
	AIN3	0.726	0.85	
	AIN4	0.711	0.854	
	AIN5	0.718	0.852	
Feedback intelligence	FIN1	0.789	0.927	0.935
	FIN2	0.838	0.917	
	FIN3	0.81	0.922	
	FIN4	0.855	0.914	
	FIN5	0.838	0.917	
Perceived ease of use	PEU1	0.699	0.823	0.859
	PEU2	0.689	0.827	
	PEU3	0.703	0.822	
	PEU4	0.729	0.811	
Perceived usefulness	PUS1	0.733	0.877	0.897
	PUS2	0.731	0.877	
	PUS3	0.738	0.875	
	PUS4	0.74	0.875	
	PUS5	0.782	0.866	
Initial trust	INT1	0.795	0.888	0.912
	INT2	0.815	0.881	
	INT3	0.801	0.886	
	INT4	0.792	0.89	
Consumer participation behavior	PAR1	0.674	0.822	0.853
	PAR2	0.673	0.823	
	PAR3	0.71	0.807	
	PAR4	0.722	0.802	
Consumer citizenship behavior	CIT1	0.836	0.929	0.941
	CIT2	0.912	0.905	
	CIT3	0.839	0.928	
	CIT4	0.847	0.926	

The AVE and combined reliability results of this study are shown in Table 6. The standardized factor loadings of all nine latent-variable items were >0.5, with AVE values exceeding 0.5 and CR values above 0.7, indicating good representativeness of the latent variables and satisfactory research convergent validity.

**Table 6:** Convergence validity test results.

Path			Coefficient of load	AVE	Combination reliability
GIN1	←-	Guidance intelligence	0.866	0.7675	0.9296
GIN2	←-		0.889		
GIN3	←-		0.867		
GIN1	←-		0.882		
RIN1	←-	Recognition intelligence	0.793	0.7272	0.914
RIN2	←-		0.831		
RIN3	←-		0.921		
RIN4	←-		0.861		
AIN1	←-	Analysis intelligence	0.771	0.5934	0.8794
AIN2	←-		0.749		
AIN3	←-		0.791		
AIN4	←-		0.771		
AIN5	←-		0.769		
FIN1	←-	Feedback intelligence	0.827	0.7435	0.9354
FIN2	←-		0.872		
FIN3	←-		0.847		
FIN4	←-		0.888		
FIN5	←-		0.876		
PEU1	←-	Perceived ease of use	0.767	0.6044	0.8593
PEU2	←-		0.762		
PEU3	←-		0.772		
PEU4	←-		0.808		
PUS1	←-	Perceived usefulness	0.784	0.6347	0.8967
PUS2	←-		0.78		
PUS3	←-		0.789		
PUS4	←-		0.791		
PUS5	←-		0.838		
INT1	←-	Initial trust	0.846	0.7243	0.9131
INT2	←-		0.867		
INT3	←-		0.853		
INT4	←-		0.838		
PAR1	←-	Consumer participation behavior	0.738	0.5938	0.8538
PAR2	←-		0.744		
PAR3	←-		0.788		
PAR4	←-		0.81		
CIT1	←-	Consumer citizenship behavior	0.842	0.7599	0.9265
CIT2	←-		0.964		
CIT3	←-		0.842		
CIT4	←-		0.832		

This study analyzes discriminant validity using the AVE values for each latent variable, with the results presented in Table 7. There were significant correlations among the nine variables (*p* < 0.01), with all pairwise correlation coefficients being smaller than the square root of AVE, indicating that the variables exhibit both correlation and independence, demonstrating good discriminant validity and meeting the research requirements.

**Table 7:** Discrimination validity test results.

Variable	FIN	AIN	RIN	GIN	PEU	PUS	INT	CIT	PAR
FIN	0.7675								
AIN	0.36**	0.7272							
RIN	0.664**	0.316**	0.5934						
GIN	0.639**	0.276**	0.683**	0.7435					
PEU	0.563**	0.379**	0.578**	0.567**	0.6044				
PUS	0.576**	0.381**	0.615**	0.566**	0.455**	0.6347			
INTt	0.248**	0.166**	0.260**	0.247**	0.316**	0.314**	0.7239		
CIT	0.214**	0.176**	0.149**	0.163**	0.166**	0.144**	0.451**	0.5938	
PAR	0.167**	0.244**	0.357**	0.266**	0.186**	0.285**	0.265**	0.169**	0.7599
AVE value	0.7675	0.7272	0.5934	0.7435	0.6044	0.6347	0.7243	0.5938	0.8007
AVE square root	0.8761	0.8528	0.7703	0.8623	0.7774	0.7967	0.8511	0.7706	0.8948

**Represents *p*-values less than 0.01; diagonal lines indicate AVE.

### 5.3 Model path test

**Figure 2:**
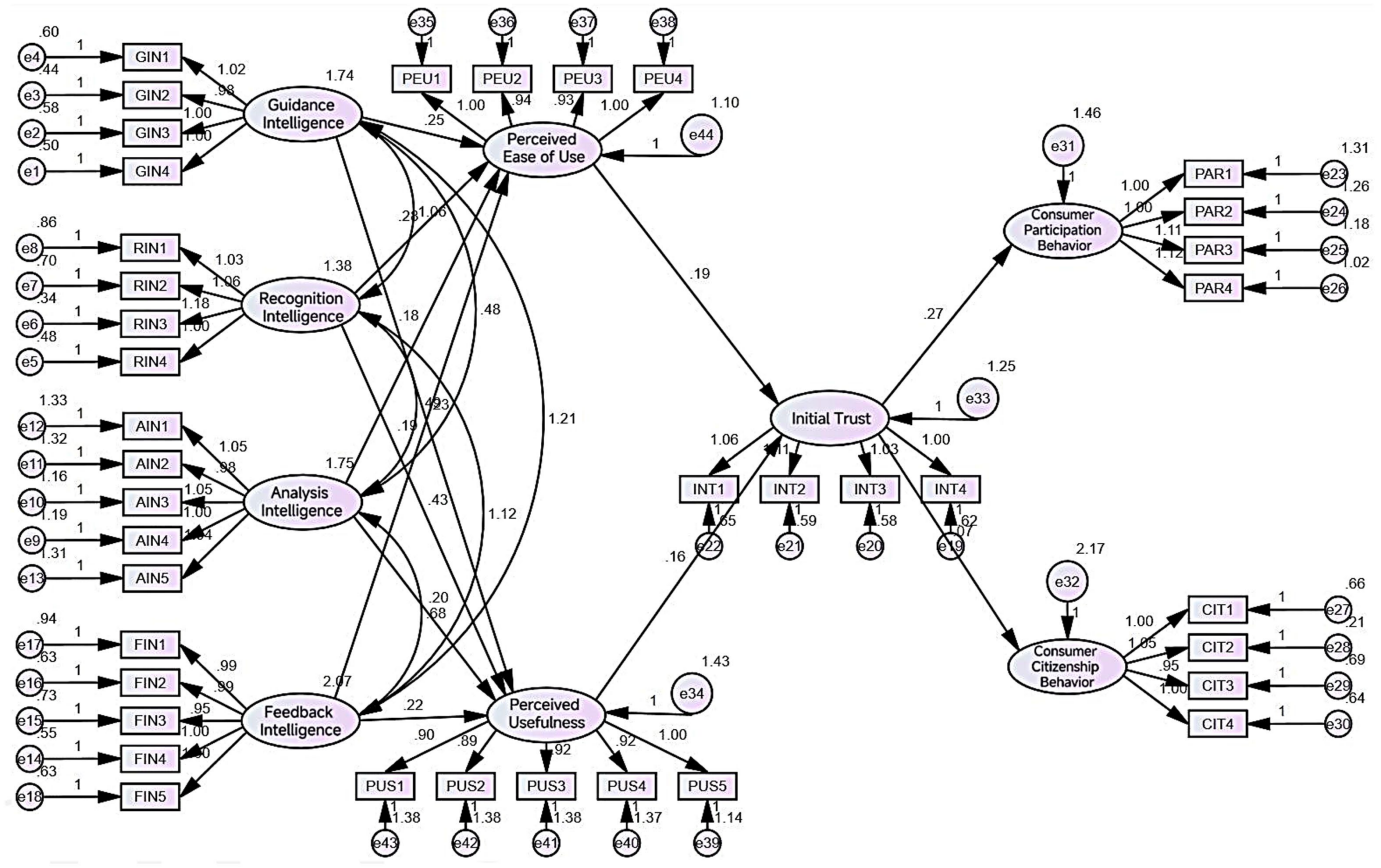
Results of the structural model test.

In this study, the research model with nine variables was constructed by using AMOS24.0 software, as shown in Figure 2:

The test results of the goodness-of-fit indicators for the research model are as follows: X^2^/df = 1.284 < 3; RMSEA = 0.029 < 0.08; GFI = 0.889 > 0.8; NFI = 0.913 > 0.9; CFI = 0.979 > 0.9; IFI = 0.979 > 0.9; TLI = 0.980 > 0.9. All the above indicator results show good fitness. Based on the goodness-of-fit indicator test results, the research model has a good fit, and no modifications are required.

This study tested the hypothesis relationship H1-12 and verified the model path in Figure 2. The specific test results are shown in Table 8.

**Table 8:** Path analysis results.

Path			Nonstandardized technique coefficient *α*	Standardization coefficient β	S.E.	C.R.	*P*
GIN	→	PEU	0.249	0.234	0.081	3.083	0.002
GIN	→	PUS	0.231	0.186	0.089	2.594	0.009
RIN	→	PEU	0.281	0.235	0.095	2.971	0.003
RIN	→	PUS	0.429	0.307	0.105	4.072	***
AIN	→	PEU	0.18	0.17	0.059	3.034	0.002
AIN	→	PUS	0.202	0.163	0.065	3.096	0.002
FIN	→	PEU	0.191	0.196	0.073	2.624	0.009
FIN	→	PUS	0.222	0.195	0.081	2.754	0.006
PUS	→	INT	0.161	0.219	0.047	3.421	***
PEU	→	INT	0.186	0.217	0.056	3.316	***
INT	→	PAR	0.272	0.262	0.064	4.235	***
INT	→	CIT	0.074	0.060	0.072	1.030	0.303

*Indicates *P* < 0.05, **indicates *P* < 0.01, and ***indicates *p* < 0.001.

Based on the analysis of the above table, this study arrives at the following research hypothesis conclusions:

Among H1-12, only H12, which posits that consumers’ initial trust positively influences consumer citizenship behavior, is not supported; all other hypotheses are validated.

### 5.4 Analysis of model adjustment effect

This study employed the Process-Bootstraping method (5,000 bootstrap samples) in SmartPLS4, using technophobia as a moderating variable to examine its effects across two groups (perceived ease of use/perceived usefulness and initial trust). Following Hair et al. (2010) criterion (*t* > 1.96 or *p* < 0.05), the results are shown in Table 9. The moderation effect of technophobia on perceived usefulness initial trust was not significant (H14 not supported), while the moderation effect on perceived ease of use-initial trust was statistically significant (H13 supported).

**Table 9:** Test results of the moderating effect.

Path	Path coefficient *β*	*T* value	*P*-value
Technophobia negatively affects (Perceived Usefulness → Consumer Initial Trust.)	0.007	0.167	0.867
Technophobia negatively affects (Perceived Ease of Use → Consumer Initial Trust.)	−0.121	2.192	0.028

**Figure 3:**
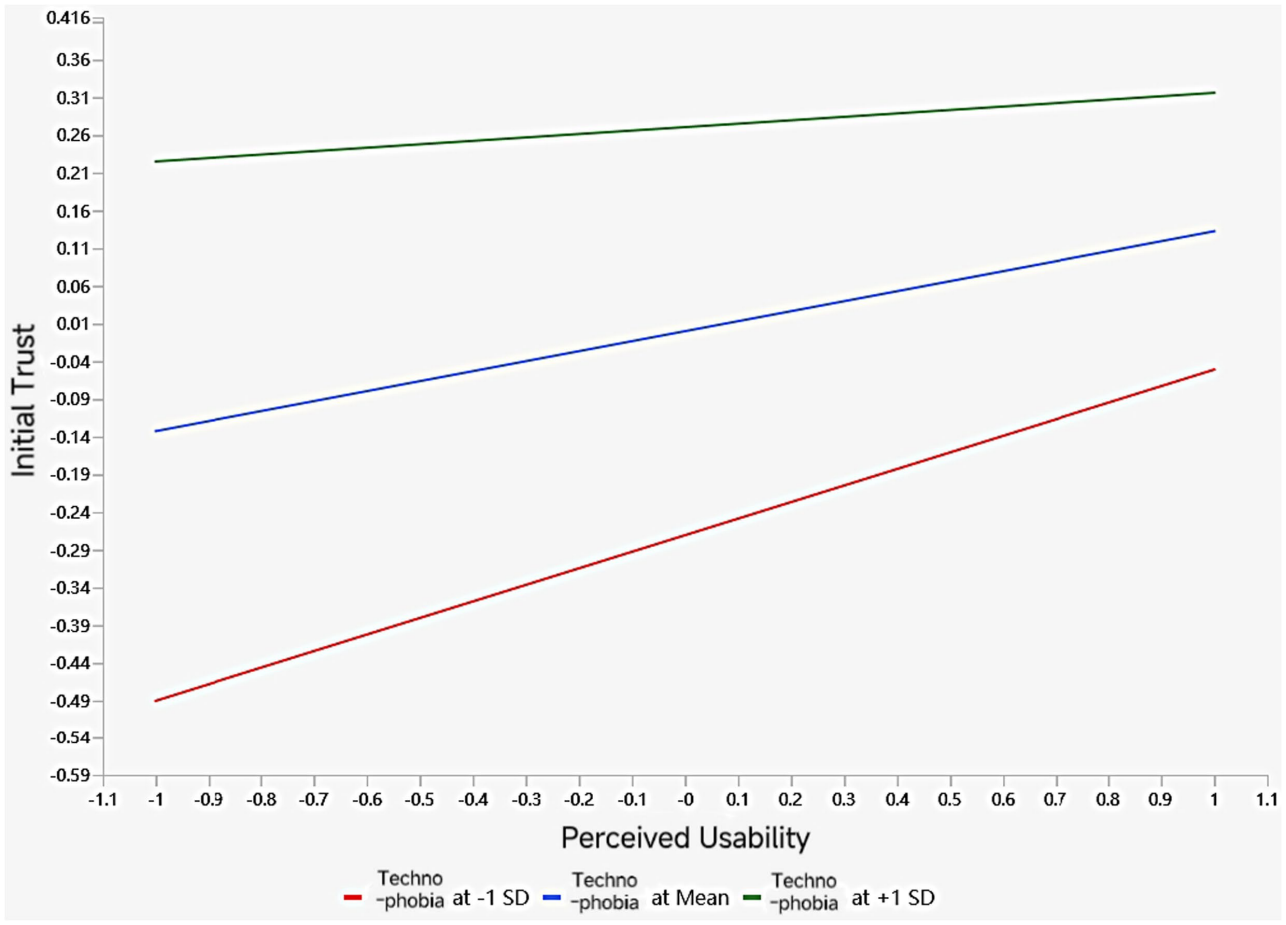
Results of simple slope analysis for moderating effect.

To examine the statistically significant moderating effect, we analyzed the model using the Process-Bootstraping method in SmartPLS4. The simple slope results are shown in Figure 3. Participants with low technophobia (at-1SD) demonstrated stronger predictive power than those with high technophobia (at +1 SD), indicating that technophobia weakens the predictive effect of perceived ease of use on initial trust. Therefore, Hypothesis H13 is supported.

## 6 Conclusion and implications

### 6.1 Research conclusions

This study confirmed the influence pathways and structural models of human–computer interaction intelligence embodied in AI virtual anchors within the context of technophobia. Of the 14 hypotheses, 12 received support, whereas two did not.

This study found that Hypothesis H12 (“Consumers’ initial trust positively affects consumers’ citizenship behavior”) was not supported. One explanation is that initial trust represents the earliest stage of trust formation (McKnight et al., 2002), dominated by preliminary expectations rather than deep participation. In contrast, consumers’ citizenship behavior entails discretionary actions aimed at improving product or service quality (Gruen, 1995). First, from the perspective of the match between trust levels and behavioral thresholds, initial trust is essentially “cognitive-level trust” and falls under the category of in-role behavior, which can only reduce the psychological barriers to shallow participation behaviors such as asking questions and leaving comments. In contrast, citizenship behavior belongs to “affective-behavioral deep co-creation” and is an extra-role behavior, which requires support from higher-level trust such as emotional connection and identity recognition (Bove et al., 2009). The transition of trust from the cognitive to the affective level cannot be achieved merely through initial contact. Second, when AI virtual anchors first appeared, their limited intelligence generated negative press coverage. Van Der Meer and Brosius (2024) empirically demonstrated that such negative news reduces audiences’ willingness to share information and increases their skepticism. Consequently, even when consumers believed AI virtual anchors could serve them, they remained reluctant to endorse them and showed low tolerance for potential shortcomings.

This study also found that Hypothesis H14 (“Technophobia negatively moderates the relationship between perceived usefulness and consumers’ initial trust”) was not supported. From the perspective of the dimensional specificity of technophobia, technophobia encompasses sub-dimensions such as fear of employment substitution, fear of operational complexity, and fear of technological out-of-control. A plausible reason is that technophobia primarily reflects individuals’ fears of job displacement by new technologies, especially by AI (Jia, 2019). Although AI virtual anchors possess utilitarian capabilities that could threaten human anchors, the sampled consumers may not have perceived their own employment to be at risk; as a result, technophobia did not interfere with the formation of initial trust. Perceived usefulness corresponds to the instrumental value of virtual anchors and has a very weak correlation with consumers’ perceived employment threats, whereas perceived ease of use is directly linked to the fear of operational complexity. This determines that technophobia cannot interfere with the “perceived usefulness-initial trust” link. Meanwhile, the formation of perceived usefulness is based on the objective functional values of AI virtual anchors, such as 24*7 service and precise product matching. Such values are irreplaceable and represent rational value judgments, which are not easily disturbed by subjective emotions like technophobia. In contrast, perceived ease of use depends on the subject’s subjective operational experience. Factors such as age and educational background may create difficulties in learning and using new technologies, leading to a sense of rejection toward new technologies among subjects (Ilona et al., 2018). Moreover, technophobia is also associated with the difficulties and uncertainties of accessing new technologies and new things (Liu, 2011), rather than the actual utility of new technologies and new things. Thus, consumers can acknowledge the usefulness of AI virtual anchors while simultaneously experiencing unease about potentially complex operations; this logic explains why Hypothesis H13, which posits an adverse moderating effect of technophobia on the relationship between perceived ease of use and consumers’ initial trust, was supported.

### 6.2 Research contributions

The core theoretical contribution of this study focuses on the trust formation mechanism and value co-creation pathways in human-computer interaction scenarios from the perspective of technophobia, particularly highlighting the unique moderating role of technophobia in the TAM-trust relationship. This innovative perspective provides a new entry point for research in related fields, specifically manifested in the following three aspects:

First, it extends the applicability and depth of the TAM. This study extends it to the context of “dynamic human-computer interaction agents” such as AI virtual anchors, systematically validating the complete transmission chain of “e-commerce virtual anchor intelligence → perceived usefulness/perceived ease of use → initial trust → value co-creation behavior.” By subdividing intelligence into four independent dimensions—guidance, recognition, analysis, and feedback—the study reveals the differential impact of these dimensions on perceptual variables, thereby enhancing the TAM model’s explanatory power at a finer granularity in AI scenarios.

Second, it clarifies the boundary moderating value of technophobia and enriches its application scenarios. This study is the first to empirically demonstrate that technophobia is not a generalized negative psychological factor that interferes with trust formation but rather specifically moderates the critical boundary condition of “perceived ease of use → initial trust.” By triggering consumer concerns about operational complexity and behavioral hesitation, technophobia weakens the conversion efficiency of ease-of-use perceptions into initial trust, while having no significant impact on the “perceived usefulness → initial trust” transmission. This finding clarifies the mechanism of technophobia in human-computer interaction trust formation, fills a research gap in technophobia theory within the context of e-commerce virtual anchors, and provides a core theoretical explanation for understanding “why some consumers recognize the value of e-commerce virtual anchors but are reluctant to establish trust.”

Third, it refines the mechanism by which initial trust differentially affects value co-creation. This study finds that initial trust can only significantly drive consumer participation behaviors (e.g., low-threshold interactions such as asking questions or leaving comments), but cannot trigger citizenship behaviors (e.g., proactive advocacy or defect feedback, which require deeper participation). It confirms an essential difference in the depth of trust required for the two dimensions of value co-creation—citizenship behaviors demand sustained and profound trust accumulation, which cannot be supported solely by the initial trust formed during first contact. This conclusion enhances the theoretical understanding of the relationship between trust and value co-creation, providing an important reference for future research to distinguish the behavioral effects of trust at different levels.

### 6.3 Management implications

Based on the research findings, this study provides actionable, practical strategies for e-commerce enterprises, live streaming platforms, and regulatory authorities, focusing on three core directions: “Optimizing virtual anchor AI functions, alleviating consumer technophobia, and tiered guidance for value co-creation behavior.”

First, E-commerce Enterprises: Prioritize upgrading core AI capabilities to enhance consumer perception and experience precisely. E-commerce enterprises need not pursue comprehensive AI upgrades; instead, they should focus on dimensions with greater impact on consumer perception variables, such as recognition intelligence and feedback intelligence. Strengthen demand recognition algorithms to accurately interpret consumer inquiries, optimize human-like feedback phrasing and response speed to reduce operational concerns, and efficiently boost initial consumer trust through targeted investments.

Second, E-commerce Enterprises, Live Streaming Platforms, and Regulatory Authorities: Collaborate to alleviate consumer technophobia and lower the entry barrier for first-time users. E-commerce enterprises may adopt a hybrid live streaming model featuring “human anchors + virtual anchors” as a transitional approach. Live streaming platforms can create simplified interaction guides (e.g., “One-Click Question Tutorials”) and organize experiential activities, such as virtual anchor sales competitions. Regulatory authorities can disseminate information on the principles of virtual anchor technology and its security mechanisms through official channels. This tripartite cooperation aims to mitigate consumer fear of unfamiliar technology and operational hesitations.

Third, E-commerce Enterprises and Live Streaming Platforms: Implement tiered guidance for consumer value co-creation that aligns with consumers’ initial trust levels. During the initial trust phase, e-commerce enterprises and live streaming platforms should focus on low-barrier participation behaviors (e.g., asking questions, commenting, liking) by using interactive prompts and coupon incentives to encourage participation. As consumers build sustained trust, deeper citizenship behaviors—such as defect feedback and proactive promotion—can be encouraged through point rewards and exclusive benefits, thereby avoiding consumer resistance to excessive demands.

### 6.4 Research limitations and future directions

First, there is a lack of tracking analysis on the evolution of trust after the first contact. This study relied on a cross-sectional design that captures only static data at the “first-contact” moment; it does not trace how initial trust evolves into sustained trust. Because the mechanism linking first contact to long-term behavior remains unclear, future research should adopt longitudinal designs to map the trajectory from initial to sustained trust. Such studies could track the same consumer cohorts, examine how sustained trust shapes value co-creation behaviors, and provide empirical guidance for the long-term deployment of AI virtual anchors.

Second, other potential interfering variables in the first-contact scenario have not been considered. This study introduced technophobia as a moderating variable, but individual characteristics of consumers (such as AI technology involvement and online shopping experience) and external characteristics of e-commerce virtual anchors (such as anthropomorphism degree and voice style) that may significantly affect the formation of initial trust during the first contact were not taken into account. In future research, the researchers will incorporate key interfering variables in the first-contact scenario to improve the theoretical model and enrich its interpretive dimensions.

Finally, the measurement of technophobia did not distinguish between the differences between “mild concerns” and “severe resistance.” The present study treated technophobia as a single construct and did not distinguish between “mild concerns” (e.g., worry about operational unfamiliarity) and “severe resistance” (e.g., outright aversion to AI). The moderating effect of perceived ease of use on initial trust may vary slightly across these levels, suggesting that the current understanding of technophobia’s moderating mechanisms could be refined. Future research should disaggregate technophobia by severity and test level-specific moderating effects, enabling e-commerce companies to design tailored fear-relief strategies for consumers with differing degrees of technophobia.

## Data Availability

The original contributions presented in the study are included in the article/supplementary material, further inquiries can be directed to the corresponding author/s.
